# Ovarian Sensitivity Index (OSI): Validating the Use of a Marker for Ovarian Responsiveness in IVF

**Published:** 2019

**Authors:** Vikas Yadav, Neena Malhotra, Reeta Mahey, Neeta Singh, Alka Kriplani

**Affiliations:** -Department of Obstetrics and Gynecology, AIIMS, New Delhi, India

**Keywords:** Agonist protocol, AMH (Anti mullerian hormone), IVF (In vitro fertilization), Ovarian sensitivity index (OSI)

## Abstract

**Background:**

In this study, an attempt was made to validate the use of OSI as a measure of ovarian response during IVF treatment and to correlate OSI with age and BMI and other measures of ovarian response such as AMH, antral follicle count (AFC), total dose of administered gonadotrophins, and duration of stimulation.

**Methods:**

This study was a retrospective comparative cohort one. The study included a total of 2150 women who underwent the first IVF cycle between January 2008 and December 2017 at our center using long-agonist protocol. Patients were divided into four subgroups according to the circulating AMH level: below the 25th percentile (AMH 0.25–1.1 *ng/ml*, subgroup A), between 25th and 50th percentiles (AMH 1.2–1.6 *ng/ml*, subgroup B), between the 50th and 75th percentiles (AMH1.7–2.6 *ng/ml*, subgroup C), and above the 75th percentile (AMH 2.7–8.5 *ng/ml*, subgroup D). Qualitative data were analyzed by Chi-square or Fisher’s exact test. The p<0.05 was considered statistically significant.

**Results:**

The four subgroups formed on the basis of the AMH level did not significantly differ for age, BMI and infertility duration. OSI was significantly correlated to age (r=0.167; p=0.001), and has negative correlation with AFC (r=−0.236, p=0.001) and AMH levels (r=−0.123, p=0.001). Multiple linear regression analysis was done on OSI with other independent variables such as age, BMI, AFC, AMH. Analysis showed that approximately 8% variation in the value of OSI can be attributed to these variables with the highest correlation with antral follicle count.

**Conclusion:**

The present study showed that OSI appears to be a highly reliable index of ovarian responsiveness to recombinant FSH and can be useful to estimate the FSH dose.

## Introduction

Controlled ovarian stimulation (COS) is a key component of assisted reproduction technologies (ARTs) and involves the development and growth of multiple follicles under the influence of gonadotropins. In essence, the multi-follicular stimulated cycle yields an increased number of oocytes improving pregnancy rate in women undergoing IVF/ICSI by increasing the number of embryos available not only for extended embryo culture but also for allowing selection of best quality embryos for transfer (1). Reinforcing the increased oocyte retrieved after COS, studies have demonstrated that ovarian response is related to the live birth rates (LBRs), with the optimal number of oocytes varying between 8 to 18 suggesting a high probability of live birth after embryo transfer (2). While the goal during any COS is to get the optimal number of oocytes, it may not be achievable in all women. Women may respond variably to COS as high (Hyper), normo (Normal) or poor (Low) responders; the decision to categorize women into each group is dependent firstly on ovarian reserve assessments done prior to the start of the cycle and secondly on the number of oocytes recovered after COS. The assessment of ovarian reserves involves a battery of variables including age, antral follicle counts (AFCs), hormonal tests including FSH, E2 and anti-mullerian hormone (AMH). Over the last decade, a number of clinical, endocrine and ultrasound parameters have been proposed as markers of ovarian reserve and predictors of ovarian responsiveness, but these have limited predictability (2, 3). Currently, the anti-mullerian hormone (AMH) is considered a promising marker corresponding to the number of small antral follicles with superior inter-cycle reproducibility compared with FSH and AFC (4–6). Responsiveness to gonadotropins stimulation has important implications on treatment success in *in vitro* fertilization (IVF) (7). Of all the tests, AMH and AFC are the most recommended ones in grouping women into response categories. This strategy using ovarian reserve markers is able to identify predictable poor responders and may hold a good predictive value in a large population but may not hold true in an individual. It is therefore not surprising to find women with normal ovarian reserves to have a poor or sub-optimal response and vice versa to COS. The other strategy of assessing response by the number of growing follicles, E2 levels and the number of oocytes retrieved is a more relevant strategy in terms of defining ovarian “response” rather than “reserves”. However, this methodology has lacked uniformity and there will be a subset of women who despite normal ovarian reserve tests may demonstrate a poor or sub-optimal response. Therefore, the response to COS with the number of oocytes recovered is yet another approach to define response. Usually, the number of oocytes retrieved is considered as the main outcome measure of ovarian re-sponsiveness to gonadotrophin stimulation. POSEI DON group (8) suggests that stimulation should be tailored according to the age related embryo/blastocyst aneuploidy rate with the intention to retrieve the number of oocytes necessary to obtain at least one euploid embryo for transfer in each patient. Normal responders are those who produce 10–15 oocytes (9). There is no single test that correlates with pregnancy, so it is not clear how to prognosticate patients. AMH and AFC have been shown to have a good correlation with each other. But the problem with these tests is related to the fact that although AFC may be more machine and operator dependent and could have higher inter-operator variability, AMH despite the ease of testing has high variability depending on assays used. Moreover, there are women who show discordancy between AMH and AFC challenging decisions during IVF treatment (5, 6).

It has been observed that both absolute numbers of oocytes retrieved and total gonadotrophin dose are important measures of ovarian responsiveness and a ratio of them is a better representation of ovarian responsiveness rather than either parameters on its own. The ratio termed as ovarian sensitivity index (OSI) was first proposed by Biasoni et al. (10). OSI has good correlation with AMH and AFC, which are considered best markers of ovarian responsiveness. The use of OSI in place of number of retrieved oocytes as the measure of ovarian responsiveness would be more appropriate where different patients are subjected to different daily dosages of gonadotrophins. So OSI is not influenced by inter-individual variability or assay methods.

This retrospective analysis was carried out to validate the use of OSI as a measure of ovarian response during IVF treatment in predicting response in our settings. Furthermore, OSI was correlated with age and BMI and other measures of ovarian response such as AMH, antral follicle count (AFC), total dose of administered gonadotrophins, and duration of stimulation and the number of retrieved oocytes.

## Methods

The study included a total of 2150 women who underwent the first IVF cycle between January 2008 and December 2017 at All India Institute of Medical Sciences, Delhi using long-agonist protocol, excluding those who used donor oocytes, individuals with high risk for OHSS and blastocyst transfer. Only the first treatment cycle of each subject was included in the study.

### Patients were divided into four subgroups according to the circulating AMH level:

below the 25th percentile (AMH 0.25–1.1 *ng/ml*, subgroup A), between the 25th and 50th percentiles (AMH 1.2–1.6 *ng/ml*, subgroup B), between the 50th and 75th percentiles (AMH 1.7–2.6 *ng/ml*, subgroup C), and above the 75th percentile (AMH 2.7–8.5 *ng/ml*, subgroup D). There were 588 patients in subgroup A, 492 in subgroup B, 558 in subgroup C and 512 in subgroup D.

Clinical data was retrieved from computerized clinical data base of the center as well besides review of patients’ clinical records. Ethical approval was obtained from concerned authority.

A transvaginal scan was performed using a 6.5 *MHz* vaginal probe to count the total number of antral follicles ranging from 2 to 10 *mm*. All ultra-sound scans were performed by reproductive medicine specialist in IVF unit.

### Ovarian stimulation protocol:

All patients received “long” gonadotropin releasing hormone (GnRH)-agonist protocol. Leuprolide (Leupride, Bayer Zydus, India) subcutaneous injection 0.5 *mg* was started in midluteal phase in preceding cycle for 8–12 days until complete pituitary desensitization was documented by ultrasound examination and estradiol measurement. Pituitary desensitization was considered when serum estradiol values were less than 50 *pg/ml*, and no residual cyst was present on a transvaginal scan. Ovarian stimulation was then started by administering recombinant follicle stimulating hormone (r FSH; Gonal F, Merck–Serono, Mumbai, India) at a daily dose of 150–450 *IU* which was individually established according to age, body mass index, basal FSH and AFC. The initial dose of gonadotrophin stimulation was decided based on the baseline of AFC and AMH (AFC≥15: 150 *IU* per day; AFC between 6 and 14: 300 *IU*; AFC≤5: 300–450 *IU*; AMH>3.5 *ng/ml* 150 *IU/day*, 2,5–3.5 *ng/ml*: 225 *IU/day*, 2.0–2.5 *ng/ml*: 300 *IU/day*;1.5–2.0:300–375 *IU* and AMH≤1.5:375–450 *IU*).

Ovarian response to stimulation was monitored by transvaginal ultrasound examination and estradiol measurement. From day 8, the rFSH dose was adjusted according to ovarian response. When two or more leading follicles reached more than 17 *mm* diameter, a subcutaneous injection of 250 microgram recombinant human chorionic gonadotrophins (hCG) (Ovitrelle, Merck–Serono, Mumbai, India) was administered. Ovum pick up was scheduled 36 *hr* after the hCG trigger and was performed by an experienced operator. A single lumen aspiration needle (Cook, Sydney, Australia) was used in all ovum pick-ups and follicular fluid was immediately given to the embryologist for oocyte identification and retrieval.

AMH measurement for all patients was done between days 2 to 5 of the menstrual cycle; venous blood sample was taken approximately within 2 months before the scheduled IVF treatment. AMH was measured using commercially available enzyme, immunoassay kit (ImmunotechBeckmamm Coulter, Webster, TX, USA).

Ovarian sensitivity index (OSI) was calculated by dividing the total administered rFSH dose by the number of oocytes retrieved at OPU to obtain the FSH-to-retrieved oocyte ratio (10).

Excel sheet was made and age, BMI, AFC, total FSH dose, stimulation length (Days), oocytes retrieved were noted. OSI was calculated by dividing the total administered rFSH dose by the number of oocytes retrieved at OPU to obtain the FSH to retrieved oocyte ratio.

### Statistical analysis:

Data are expressed as mean±SD or counts and percentages. Qualitative data were analyzed by means of Chi-square or Fisher’s exact test. The normality assumption of the quantitative measures was verified by Shapiro-Wilk test and significance of between-group differences was assessed using ANOVA. Pairwise comparisons of the groups were performed with Bonferroni’s adjustment for multiple comparisons. Pearson correlation was used to test the relationship between OSI and patients’ age, BMI, AFC and circulating AMH. To analyze the correlation between OSI and AMH, a linear regression analysis adjusted by age was also performed. The p<0.05 was considered statistically significant.

### Ethics Committee:

Not applicable, retrospective data. As per local protocol, ethical approval. And individual patient consents were not required to analyze anonymized hospital data.

### Research involving Human Participants and/or Animals:

Retrospective study and only datawas used with permission of institute record section available for academic purpose.

### Informed consent:

Not applicable; Retrospective study.

## Results

A total of 2150 patients were included in the present study. The mean±SD of age, BMI and length of stimulation was 31.4±3.6 years, 24.9± 3.8 *kg/m*^2^ and 11.3±1.7days, respectively. The mean total dose of recombinant FSH was 3297± 1092 *IU/day*. On average, 9.7±5.2 oocytes were retrieved from all these patients.

The four subgroups formed on the basis of the AMH level did not significantly differ for age, BMI and infertility duration (Table 1). AFC was progressively higher from subgroup A (10.9±3.8) to subgroup D (14.1±4.4). Furthermore, mean AFC in subgroup D was significantly higher than the other subgroups (p<0.0001). The average number of retrieved oocytes in subgroup D (11±5.5) was significantly higher than the one in subgroup A (8.5±4.5), but not significantly different compared to subgroups B and C. The total administered rFSH dose and the OSI showed the similar trend that was observed in AFC indicating decreasing trend from subgroup A to subgroup D and very prominently lower in subgroup D than in the other three subgroups (p<0.001) (Table 2).

**Table 1.** Characteristics of patients subgrouped according to circulating AMH levels

	**Subgroup A n=588**	**Subgroup B n=492**	**Subgroup C n=558**	**Subgroup D n=512**	**p-value**	**Significant comparison**
**AMH (*ng/ml*)**	0.24–2.30	2.31–3.03	3.04–4.20	>4.20		
**Age (years)**	31.7±3.8	32±3.6	31.1±3.6	30.6±3.5	0.001	1 *vs.* 3,4; 2*vs.* 3,4
**BMI (*kg/m^2^*)**	25.2±3.7	25.3±3.9	24.6±3.7	24.9±3.8	0.001	41*vs.* 3,4 ; 2*vs.* 3,4
**AFC**	10.9±3.8	12.1±4.8	12.2±3.8	14.1±4.4	0.001	1 *vs.* 2,3,4; 2*vs.* 4; 3*vs.* 4
**Total dose of FSH (*IU*)**	3550.9±1103.4	3361.3±1148.1	3296±1036	2946.8±993.3	0.001	1*vs.* 2,3,4 ;2*vs.* 4 ; 3*vs.* 4
**Stimulation length (days)**	11.4±1.7	11.4±1.8	11.2±1.6	11.1±1.5	>0.05	NS
**No. of oocytes retrieved**	8.5±4.5	9.1±4.8	10.2±5.4	11±5.5	0.029	1 *vs.* 3,4; 2*vs.* 3,4
**Ovarian sensitivity index (*IU*)**	616.4±688.5	500.2±517.5	432.1±353.5	365.2±321.4	0.001	1 *vs.* 2,3,4; 2*vs.* 4

AMH: Antimullerian hormone; BMI: Body mass index; AFC: Antral follicle count. FSH: Follicle stimulating hormone

**Table 2.** Correlation between ovarian sensitivity index (OSI= FSH units per retrieved oocyte) and patient’s age, BMI, antral follicle count (AFC) and circulating AMH levels

**Study parameters**	**r**	**p**
**Age**	0.167	0.001
**BMI**	0.034	0.118
**AFC**	−0.236	0.001
**AMH**	−0.123	0.001

### Bivariate correlation analysis between study variables:

OSI was significantly correlated to age (r= 0.167; p=0.001), and similar observation could not be obtained for BMI. OSI was also showed a significant negative correlation with AFC (r=−0.236, p=0.001) and AMH levels (r=−0.123, p= 0.001). Multiple linear regression analysis was done on OSI with the other independent variables such as age, BMI, AFC, AMH. Analysis showed that approximately 8% variation in the value of OSI can be attributed to these variables with the highest correlation with antral follicle count (Table 3).

**Table 3.** Multiple linear regression analysis on OSI with different variables

	**Regression coefficient**	**Std. error**	**Sig.**	**R**^2^
**Constant**	225.785	114.810	0.049	7.8%
**AGE**	18.653	2.921	0.000	
**BMI**	.407	2.807	0.885	
**TOTAL AFC**	−23.666	2.474	0.000	
**AMH**	−13.526	4.741	0.004	

## Discussion

In the last decade, serum AMH measurement has emerged as one of the best markers of ovarian reserve (11). It has been a biomarker to predict the response to COS in terms of quantity of oocytes retrieved (5, 6), and lately even related to live birth in IVF cycle (12). AMH is a dimeric glyco-protein belonging to the transforming growth factor –beta (TGF-β) superfamily, produced by granulosa cells of small pre-antral follicles (13). Also, its serum levels show a very low inter-and intra-cycle variability and are independent of the menstrual cycle phase (14).

AFC is equipment and operator dependent and has been shown to have important inter-observer variations (15), but in our study, it was performed in a single center with only few experienced operators. In combination, AMH and AFC are among the best ovarian response markers for prediction of ovarian response in IVF treatment.

Various models use these variables (Age, BMI, AMH, AFC, FSH) separately or in combination to reach an acceptable, even if not optimal, level of accuracy in estimating ovarian responsiveness to exogenous gonadotropins (16). Properly choosing the initial dose of gonadotropins avoids a poor oocyte yield in IVF. In most IVF centers, dose of gonadotropins is usually based on age, BMI, basal FSH level, antral follicle count and most importantly AMH.

In our study, age and BMI had significant correlation with ovarian response, similar to other studies (17).

Ovarian response was studied in terms of the number of retrieved oocytes as well as OSI. OSI which refers to the number of oocytes retrieved for gonadotrophins administered is a measure of ovarian responsiveness. This ratio represents the ovarian resistance to gonadotrophins; the lower FSH dose is, the higher the ovarian sensitivity will be. The present study showed that OSI displays a strong, inverse correlation with AMH levels, and the correlation is stronger than that between AMH and total gonadotrophin dose or between total numbers of oocytes retrieved.

In our study, there is a good correlation of OSI with age and BMI, thus suggesting that these variables could be incorporated in prediction models aimed at predicting ovarian responsiveness to exogenous gonadotrophins.

The use of this ratio in our study eliminates the confounding effect of different initial doses of gonadotrophin being used based on AFC, which allows more appropriate comparison of ovarian response.

Our data was obtained using GnRH agonist leuprolide plus recombinant FSH in a classical long protocol. The current study only included those treated on the long GnRH agonist protocol in order to avoid the confounding effect of different protocols on the outcome measures. As most of our subjects were treated on the long agonist protocol during the study period, this gave a fair representation of the overall patient cohort. It must be noted that the correlation between OSI and AMH could be slightly different in case of different stimulation protocol usage. However, the short-comings of our study are the retrospective design and also the numbers. Considering that only long agonist was included with the use of rFSH the numbers could not be large as they came from a single unit. A large multicenter trial including even the antagonist and other protocols may perhaps help to set cutoff values of OSI that can point out the patient with good prognosis for future IVF and thus will help in better patient selection and more importantly counseling of the patient. Perhaps a multicenter data would have offset this drawback. While other units do use long agonist and rFSH, the problem in pooling data from other units is that there is no standard protocol with COS as some clinicians would keep doses of gonadotropins fixed while others would be flexible, titrating as per the response on follicle monitoring and estradiol levels during stimulation.

## Conclusion

The present study shows that OSI appears to be a highly reliable index of ovarian responsiveness to recombinant FSH and can be useful to estimate the FSH dose. Larger scale studies are required to conclude that OSI is used as a supporting marker to AMH for predicting the ovarian responsiveness.
